# Medical and Philosophical Intelligence

**Published:** 1813-06

**Authors:** 


### MEDICAL AND PHILOSOPHICAL INTELLIGENCE.

*ROYAL SOCIETY*.—On Thursday, the 25th of February, was read a paper on the black matter in the glands of the lungs in old persons, by Dr. Pearson. The lungs are at first light-coloured, but they become mottled about the age of 20, gradually increase in darkness, and in old persons are nearly black. Dr. Pearson examined the cause of this change, and found it owing to a quantity of charcoal contained in the glands. This charcoal, he conceives, is suspended suspended in the air, and taken in with the breath. He accounts for its absence in young persons, and in brute animals, by the state of the lymphatics; by means of which, he conceives the charcoal makes its way into the bronchial glands.

On Thursday, the 4th of March, a paper by the late Mr. Kirby Trimmer was read, on the fossils found in the neighbourhood of Brentford. This town lies on the side of the Thames, about six miles west from London. The soil, as far as it has been dug, consists of five distinct beds. The uppermost is a gravelly loam; the second, sand and gravel; the third, a calcareous loam; the fourth, sand; and the fifth, blue clay, which passes under London, and is found every where in the neighbourhood. The thickness of the clay bed is 200 feet, reckoning from the surface of level ground. Its depth on hilly grounds is greater; thus Lord Spencer, at Wimbleton, was obliged to dig a well 530 feet deep, before he got through the clay. The uppermost bed contains no fossil remains whatever. The next three contain the tusks of elephants, both African and Indian, of the hippopotamus, the horns and jaws of oxen, the horns of deer, snail-shells, and the shells of fresh-water fish; but no sea animals. The clay contains the fossil remains of sea animals alone, as echini, shells, &c. These fossils are scattered without order in the beds, and the bones must have been deposited long after the death of the animals, for no two are found contiguous in the order in which they existed in the living animal.

On Thursday, the 11th of March, there was read a description of a glass apparatus for condensing gases, by Mr. Austin. This was a modification of a condensing apparatus formerly contrived by Mr. Austin, the description of which was published in one of the volumes of the Memoirs of the Royal Irish Academy. It is needless to attempt any account of this apparatus, as we could not make it intelligible without figures.

On Thursday, the 18th of March, a paper was read by Sir Everard Home, Bart. on the formation of fat by the lower intestines. During the investigations respecting the digestive organs of animals, in which Sir Everard Home has been engaged for many years, he was gradually led to the opinion that after the chyle has been separated from the food in the smaller intestines, it undergoes a new process in the larger intestines, where animal fat is separated from it, and the residue is converted into excrementitious matter. He conceives that the situation of the food in the lower intestines is similar to that of bodies which are converted into adipocire in the ground. He gives an example of this change in Shoreditch church-yard, where several bodies were found converted into adipocire, in the course of ten years. They were buried about ten feet from the common sewer, and about two feet below it. There is always a current of water in this sewer, and at new and full moon it rises two feet higher than usual, and at that time there is water in the graves. Ambergrease is found in the lower intestines of whales: he conceives it to be formed in consequence of a disease in the animal, which prevents the fat from being absorbed as it is formed. Fat is sometimes formed, and thrown out of the human intestines. Of this Sir Everard related

 several several curious cases. He conceives that the formation of fat is owing to the action of the bile on the food in the greater intestines. Mr. Brande, on trying the experiment, found that animal muscle, digested in bile of the temperature 100° for four days, acquired the smell of excrement, and was partly changed into fat. He found that the fæces of fowls, retained in the cœcum for seven days, were converted into fat by the action of diluted nitric acid; but not the fæces in the colon. He found that human fæces, retained in the intestines for six days, contained fat which was separated by digestion in hot water. He related an instance of a child that never grew, though it took food, and was not emaciated; and which, on dissection, was found without a gall bladder, or biliary ducts. Hence he conceives that fat was not secreted, and that fat is necessary to the growth of animals. This curious theory throws a new light upon digestion, and explains several circumstances which appeared before anomalous; as the fatty concretions in the gall bladder, which are owing to the action of the bile on the mucus of the bladder.

On the 1st of March some additional observations on the tusks of the narwal, by Sir Everard Home, Bart. were read. He found in the skull of a female narwal two milk tusks, about nine inches long. Hence that animal, when full grown, would have had tusks. On sawing in two the tusk of a narwal it was found mostly hollow. This is the reason why it cannot be used, like ivory, for the purposes of art.

At the same meeting of the Society, a paper by Dr. Wells was read, giving an account of a woman, the offspring of white parents, part of whose skin was black. The name of the woman is Harriet West. She was born in Suffolk, about eight miles from the sea coast; and she is at present about 23 years of age. Her father was a footman, and died when she was very young. She is the only child of her father; but her mother, who was married a second time, has had eleven children since, all white. Her mother, when pregnant with her, got a fright by trampling on a live lobster; and to this the spots on her skin were ascribed. The whole of her body is very white, except the right shoulder, arm, and hand, which are mostly black, except a white stripe on the fore arm. The black parts are darker than in a negro. Winslow has observed, that the cuticle in negroes is black; and Dr. Wells found this the case with the black cuticle of Harriet West. From this curious case, Dr. Wells draws the following inferences:—1. The black colour of negroes does not prove them to be a distinct race of animals from the whites. 2. The black colour cannot be ascribed to the action of the sun merely, as is the common opinion. An additional proof of the fallacy of such an opinion is, that those parts of negroes which are exposed to the sun are not so black as those that are covered with clothes.

On Thursday, the 8th of March, Dr. Wells's paper was concluded. He gave his opinion about what occasioned the difference between negroes and whites. It is well known that whites are not so well able to bear a warm climate as negroes; and that they are liable to many diseases in such a situation, from which negroes are free. On the other other hand, whites are much better fitted to bear a cold climate than negroes. Suppose a colony of whites transported to the torrid zone, and obliged to subsist by their labour, it is obvious that a great proportion of them would speedily be destroyed by the climate, and the colony, in no long period of time, annihilated. The same thing would happen to a colony of negroes transported to a cold climate. Dr. Wells conceives that the black colour of negroes is not the cause of their being better able to bear a warm climate, but merely the sign of some difference in constitution, which makes them able to bear such a climate. Suppose a colony of white men carried to the torrid zone; some would be better able to resist the climate than others. Such families would thrive, while the others decayed. These families would exhibit the sign of such a constitution; that is, they would be dark: and as the darker they were, the better they would be able to resist the climate; it is obvious that the darker varieties would be the most thriving, and that the colony, on that account, would become gradually darker and darker coloured till they degenerated into negroes. The contrary would happen to negroes transported to cold climates.

Dr. Wells conceives that the woolly hair, and deformed features of the negroes, are connected with want of intellect. The negroes have been always slaves; and there is no instance of their better shaped neighbours being subject to the negroes.

The same evening a paper by I. Berzelius and Dr. Marcet, on the *alcohol of sulphur*, was begun. This substance was discovered several years ago by Lampadius, while distilling a mixture of charcoal and pyrites. He called it alcohol of sulphur from its great volatility. He conceived it to be a compound of sulphur and hydrogen. Clement and Desormes obtained it soon after by passing sulphur through red-hot charcoal; and from their analysis it appeared to be a compound of sulphur and charcoal. Berthollet, in his elaborate remarks upon carbonic oxide, and his critique upon the experiments of Clement and Desormes, revived the opinion of Lampadius; and this was confirmed by the experiments of Berthollet, jun. The subject was lately resumed by Clusel, who concluded from his experiments, that the substance was a triple compound of sulphur, charcoal, hydrogen, and azote. Thenard examined the substance anew, and found it a compound of sulphur and charcoal alone. These discordant results prevent any confidence from being put in the various experiments already made. It was to remove the doubts still hanging over the subject that Berzelius and Marcet were induced to undertake the investigation of the subject.

---

*Linnæan Society.*—On Tuesday, March 2d, a paper by Mr. Brown, Librarian to the Society, was read, on some peculiarities in the structure of seeds. All seeds have hitherto been considered as inclosed in a covering; but in some, after they have come to maturity, such covering cannot by any means be separated from the seed. Such seeds have been called *naked* seeds, and they have been divided into two species. Mr. Brown has observed two examples of seeds, not hitherto therto suspected to exist, namely, seeds absolutely destitute of a covering, from their first appearance till their state of ripeness.

On Tuesday, the 16th of March, a number of specimens of rotten wood were exhibited by Mr. Sowerby, in order to illustrate the nature of the dry rot in wood, which is occasioned by different species of fungi.

Part of a paper, by the Rev. Patrick Keith, was read, on the cotyledons of grasses. The seeds of grasses, if we do not reckon their outer coats, consist of three parts: 1. A farinaceous substance, which constitutes the greatest part of the seed, and is known by the name of *albumen*. 2. A scale lying upon the surface of the albumen, to which Gærtner has given the name of *vitellus*. 3. The *embryo*, with a kind of sheathy covering. Gærtner considered this sheathy covering as the cotyledon of the grasses. But Dr. Smith conceives the vitellus of Gærtner to be the cotyledon. The object of Mr. Keith's paper is to show that Gærtner's opinion is correct, and that the vitellus does not possess the characters of a cotyledon.

On the 6th of April the remainder of Mr. Keith's paper on the cotyledons of grasses was read. He found by examination that the sheath of the plumula never assumes the form of a true leaf; that it rises out of the ground, is at first white and transparent, and then becomes purple; and that it divides, and allows the leaf of the plumula to pass through it. The secondary shoots are also furnished with sheaths; but their structure is quite different from that of the plumula. As to the scale called *vitellus* by Gærtner, Mr. Keith conceives that it may be intended to act as a strainer to the milky food prepared in the albumen.

At the same meeting an analysis of *arragonite*, by the Rev. John Holme, of St. Peter's College, Cambridge, was read. This mineral has been long known to mineralogists, and constitutes an anomaly in the Haüyan theory of crystallization. The figure of its crystals, its specific gravity, its hardness and lustre, differ from the same properties in calcareous spar; yet its constituents, as far as chemical analysis has gone, are absolutely the same. It has been analysed by Klaproth, Bucholz, Vauquelin, Chenevix, Thenard, and Biot; but nothing different from the constituents of calcareous spar was found. Mr. Holme conceives the difference to depend upon a quantity of water chemically combined in arragonite, while calcareous spar is destitute of that constituent. He found that when exposed to heat it gives out water without decrepitating, and at the same time falls to powder. This water was found to contain no carbonic acid gas, nor was any given out under a red heat. The constituents of arragonite, according to Mr. Holme, are as follows:—

| | |
|---|---|
| Lime . . . . . . . . . . | 55·5 |
| Carbonic acid . . . . . | 43·7 |
| Water . . . . . . . . . | 0·8 |
| | 100 |

It is by no means unlikely that the proportion of water here assigned is too small; for nobody can believe that all the water present can be driven driven off at a heat below redness. Theory gives us the constituents of arragonite as follows:—

| | |
|---|---|
| Lime . . . . . . . . . | 55·50 |
| Carbonic acid . . . . . | 42·23 |
| Water . . . . . . . . | 2·27 |
| | 100 |

Now it is certainly a very odd circumstance that so small a portion of water should act in this case as the cement. The weight of an atom of anhydrous carbonate of lime is 47·9, and that of an atom of water 8·5. Hence it would appear that every atom of water in the arragonite is surrounded by eight atoms of carbonate. This might be conceived to form a cube; but never could constitute the tetrahedron which Haüy conceives to be the form of the integrant molecule of arragonite. Mr. Holme proposes to call arragonite *hydrous carbonate of lime.*

On the 20th of April a fossil chama car, filled with primitive crystals of carbonate of lime, supposed to be from Wiltshire, was exhibited by Mr. Sowerby.

A fossil turtle, from a quarry in Dorsetshire, was exhibited by Mr. Bullock. The specimen was very perfect, and exhibited the shell of the turtle almost complete. The quarry, from the pieces of stone attached to the specimen, I conceive to be limestone. Only another specimen of fossil turtle was found in this quarry, and it was broken in taking it out.

A letter from Mr. Heyne was read, giving an account of a very singular change which takes place daily in the leaves of a species of cotyledon from India, which is cultivated in our hot-houses. In the morning these leaves are as sour as the leaves of sorrel, at noon they are tasteless, and in the evening they are somewhat bitter. Mr. Heyne explains this singular change by supposing that the plant absorbs oxygen gas during the night, and forms an acid which is again decomposed during the day. Though this explanation be very unlikely to be the true one, the phenomenon certainly deserves to be particularly examined. What is the acid? and what becomes of it?

A paper by Mr. Annesley was read, giving a description of a new species of rubus, which he observed first in Wales, and afterwards in Perthshire and Aberdeenshire. It is the same which was observed by Mr. Hall at Loch Ness, and which he described in the Transactions of the Royal Society of Edinburgh. It is found likewise in Yorkshire, and many other parts of Great Britain.

---

*Imperial Institute of France.*—Account of the Labors of the French Institute for 1812.—PHYSICAL DEPARTMENT. By M. Le Chev. Cuvier, Perpetual Secretary.

*Physics and Chemistry.*—Every one knows that heat is one of the principal instruments of chemistry, and one of the greatest forces which act in its phenomena. We may consider it in itself, in its effects, and in its sources.

Count Rumford, who is continually occupied with the sciences, as far as they contribute to the good of society, has this year treated of hea heat under this last point of view, and has endeavoured with much care to determine how much heat is produced by the combustion of each substance.

To attain this object, it was necessary in the first place to have a general method of measuring exactly these quantities of heat; and when we reflect on the complicated nature of the phenomena of combustion, we must be sensible of the numerous difficulties which Count Rumford had to encounter in his attempts. It was only after a laborious investigation of 20 years that he was able to overcome them.

His principal idea was to measure the quantity of water which passes from one fixed degree of temperature to another equally fixed by the combustion of a measured quantity of each substance. The apparatus which he has contrived for this purpose consists in a prismatic and horizontal receiver of copper, in which there are two holes; one near one of the ends, to receive a thermometer, the other in the middle of the upper surface, through which water is poured in, and which is stopped by a cork. Within this receiver there is a kind of flat worm, which covers the whole bottom without touching it, and which is destined to receive the aërial products of combustion by means of a vertical funnel soldered to its orifice. This worm returns three times on itself, and its other extremity traverses horizontally the upper surface of the receiver, to which it is contiguous. The goodness of the whole apparatus depends upon the flat form of the worm, which ought to transmit to the liquid contained in the receiver all the portion of heat which it receives from the substance that is burnt.

But the receiver, when once hotter than the surrounding air, must lose a portion of the heat which it has received; and the azote of the air which has served for the combustion, being with the other products in the worm, must likewise retain a portion of that heat. To remedy these two causes of error, Count Rumford conceived the simple and efficacious idea of beginning his experiments at a determinate degree below the temperature of the ambient air, and to stop them when the water of the receiver has reached an equal number of degrees above that temperature; so that in the first part of the experiment the surrounding air and the azote furnish just as much heat to the receiver as they take from it in the last part of the experiment.

The cylindrical reservoir of the thermometer has precisely the same height as the receiver; so that it indicates precisely the mean temperature of the whole water in the receiver.

Count Rumford, by means of this apparatus, burnt successively different combustibles, taking care that the combustion was complete, that no residuum was left, and that neither smoke nor smell was emitted during the combustion. He found that a pound troy of each combustible, during its combustion, raised the heat of the following quantities of water from the freezing to the boiling point.

| | |
|---|---|
| White wax . . . . . . | 72108 lbs. troy. |
| Olive oil . . . . . . | 68900 |
| Oil of colza . . . . | 70906 |
| Alcohol . . . . . . . | 51400 |
| Sulphuric ether . . . | 61178 |
| Naphtha . . . . . . | 55900 |
| Tallow . . . . . . . | 63755 |

It

It is remarkable, that, if we admit the accuracy of the analysis of these substances made by Lavoisier, Cruikshank, De Saussure, Gay-Lussac, and Thenard; and, if we calculate the heat that would have been produced by the hydrogen and carbon which enter into their composition, if they had been burnt separately, we obtain very nearly the same results.

We cannot make the reader sensible of all the merit of these researches, unless we were to state the numerous calculations of the author, which is incompatible with the nature of our general view.

Furnished with this previous knowledge, Count Rumford passed to the quantity of heat evolved by the combustion of wood; but here the problem became more complicated. A high temperature produces numerous changes in wood. One part of its constituents is driven off, while another enters into new combinations. It was necessary, therefore, in the first place, to examine the structure of wood, the specific gravity of its solid parts, the quantity of liquids and elastic fluids which it contains in their different states, and finally what charcoal furnishes.

After having exactly dried different specimens of wood in a stove, Count Rumford obtained this singular conclusion, that the specific gravity of the solid matter which constitutes the *timber* of wood is almost the same in all trees. By the same means he determined that the woody part of oak in full vegetation is only four-tenths of the whole. Air constitutes one-fourth of it, and the rest consists in sap. Light woods have still a much less quantity of solid matter; but the season of the year, and the age of the tree, occasion considerable variations. Ordinary dry wood contains above one-fourth of its weight of water. Even the oldest wood, though in the state of timber for ages, never contains less than one-sixth of its weight of water.

Count Rumford has determined, by exact experiments, that all absolutely dry woods give from 42 to 43 per cent. of charcoal. Hence he concludes that the ligneous matter is identic in all woods. This loss, which the driest wood experiences when charred, the absolute quantity of carbon determined by Thenard and Gay-Lussac at 52 or 53 per cent., the matters which are deposited on the vessels, and finally this fact that wood too much dried, too nearly approaching to the state of charcoal, gives out less heat—all these circumstances induce Count Rumford to believe that the proper charry fibre, which he calls the woody skeleton, is surrounded by another substance, which he compares to the muscles, and which he calls *vegetable flesh*. The fire first attacks this envelope, because it contains hydrogen, which renders it more inflammable, and which contributes a great deal to the heat given out by wood.

From numerous experiments and complicated calculations, Count Rumford has drawn up a table of the quantity of water which the different woods, according to their state of dryness, can heat from the freezing to the boiling temperature. From this table it appears that the lime-tree gives out the most heat, and the oak the least, during combustion. From the same analysis it follows that the inevitable loss of heat during the charring of wood is more than 42 per cent., and by by the ordinary processes of the charcoal-makers more than 64 per cent., because they form a considerable quantity of pyrolignous acid, which consumes this great proportion of carbon. It follows, likewise, that all the charcoal furnished by any wood whatever, furnishes only one-third of the heat that is furnished by the wood itself from which it was formed.

Count Rumford conceives, likewise, that he has ascertained this important fact for chemistry, that carbon may combine with oxygen, and form with it carbonic acid, at a much lower temperature than that in which it burns visibly.

The difficulty of following this philosopher in his complicated calculations respecting the greatest intensity of heat which it is possible to produce, and on the quantity of heat evolved by the condensation of the vapor of water and alcohol, obliges us to notice only the general results. He determines, for example, that the temperature of water at the moment of its formation by the combination of oxygen and hydrogen is eight times higher than that of iron heated so as to appear red in broad day light; and that boiling-water, in passing to the state of vapor, renders latent 1040 degrees of heat, or, which comes to the same thing, that this quantity is evolved when the vapor of water is condensed.

According to the same experiments, the capacity of the vapor of water for heat diminishes with its temperature; and, from the phenomena relative to the vapor of alcohol, we may conclude that the oxygen and hydrogen which enters into the composition of this liquid are not in the state of water.

The Class had proposed, as one of its physical prizes, the determination of the capacity of oxygen gas, carbonic acid gas, and hydrogen gas, for heat. This prize has been voted to a memoir of M. M. François Delaroche and Berard. These two philosophers have not satisfied themselves with the cases proposed; they have taken a general view of the matter, and determined the specific heat of other gases; and that of air and vapor under different pressures. Among other interesting particulars, they have found that the capacity of a given mass of air increases with its bulk. Reducing all the capacities to that of water, they have drawn up the following table of their labors:

| | |
|---|---|
| Capacity of Water . . . . . . . | 1·0000 |
| Atmospheric air . . . | 0 2669 |
| Hydrogen gas . . . . | 3·2936 |
| Carbonic acid gas . . | 0·2210 |
| Oxygen gas . . , . | 0·2361 |
| Azotic gas . . . . . . | 0·2754 |
| Nitrous oxide gas . . | 0·2369 |
| Olefiant gas . . . . . | 0·4207 |
| Carbonic oxide gas . . | 0 2884 |
| Aqueous vapor . . . | 0·8470 |

Heat penetrates all bodies. It contributes essentially to their dilatation, and it is squeezed out, to use the expression, whenever they are reduced, by any operation whatever, to smaller dimensions. Thus we we know, by experiments made ten years ago at Lyons by M. Mollet, that air suddenly compressed gives out heat, and that this heat is accompanied with light. This phenomena has given origin to the convenient instrument by which tinder is kindled by the pressure of a piston.

M. Dessaignes, an ingenious philosopher of Vendome, in a memoir of which we have given an account, having subjected different gases to the same operation, obtained similar effects. Hence it was concluded, apparently with reason, that the same effects ought to appear with all the aeriform fluids. But M. de Saissy, a physician in Lyons, having repeated the experiments of M. Dessaignes, could only produce light with oxygen gas, muriatic acid gas, and common air. Oxygen gas gives the most light, muriatic acid gas comes next in order, and common air gives the least of the three. The other gases do not become luminous, except when some oxygen is mixed with them. M. de Saissy concludes from this that the aeriform fluids have not the property of giving out light by compression, except when they contain oxygen free, or feebly combined. He thinks that this fact, when once well established, will give additional probability to the opinion that heat and light are different substances.

The doctrine of M. le Comte Berthollet on the different actions which influence the definite results of chemical phenomena, depends in a great measure upon this almost general fact, that an alcali which decomposes a saline compound only deprives it of that portion of its acid to which it owed its solubility, and as soon as it becomes insoluble it precipitates, preserving the rest of its acid, and frequently taking with it even a portion of the alkali which acted on it; so that the precipitate is almost always a compound. Yet M. Taboalda had announced that the pure alkalies throw down from *corrosive sublimate* an oxide of mercury free from all acid. M. Berthollet repeated this experiment, and found that the precipitate is not pure unless more alkali be added to the solution of corrosive sublimate than is necessary to saturate all the muriatic acid present. When this is not added, the precipitate always retains a portion of acid, which varies according to circumstances. The kind of alkali is indifferent. But when the potash, for example, is completely saturated with carbonic acid, it does not decompose the corrosive sublimate. If we employ a subcarbonate, it acts till it has lost its redundant potash; but the precipitate contains both muriatic acid and potash.

The alkalies produce the same effects on the nitrate of the peroxide of mercury, and experiments made on the sulphate of alumina gave analogous results; that is to say, they confirm the law established by M. Berthollet.

The same philosopher had made experiments long ago to determine the proportion of oxygen and muriatic acid which constitute oxymuriatic acid; but Mr. Chenevix having obtained different results, M. Berthollet returned again to the same subject. He found that the light which he had at first employed as the principal agent takes only a certain quantity of oxygen from the acid, though it reduces it to a state in which its action on reactives differs little from that of common muriatic acid. Hence he concludes that this state is the first

 degree degree of oxidation of the muriatic base. Decomposing the oxy-muriatic acid completely, by means of ammonia, he found 23·64 per cent. of oxygen instead of 9·41, which was the result of his first analysis.

In one of his preceding memoirs, M. Berthollet had stated facts from which it was easy to conclude that carbureted hydrogen gases existed; but he had neglected to draw that conclusion. The analysis of olefiant gas by M. de Saussure, has set that truth in a clear light, by showing that this gas contains no oxygen, and that it is a real carbureted hydrogen gas, composed of 86 parts of carbon, and 14 of hydrogen.

Mr. Dalton, in treating of this subject in his New System of Chemistry, has endeavored to prove that hydrogen and carbon combine only in two proportions. The one gives us olefiant gas; the other the gas of marshes. He considers the gases named by Berthollet *oxycarbureted hydrogen,* as mixtures of carbureted hydrogen, carbonic oxide, and hydrogen. According to Dalton, olefiant gas, when exposed to heat, or to the action of electricity, passes to the state of the gas of marshes, by depositing one half of its carbon; and the gas of marshes, when exposed to the same action, is entirely decomposed. If we obtain a peculiar gas before that decomposition is complete, this gas is a mixture of hydrogen and the gas of marshes.

M. Berthollet has repeated these experiments with electricity; but they have not led him to the results announced by Dalton. A part only of the gas was decomposed, and that which remained undecomposed resisted the most violent action of electricity. M. Berthollet concludes, likewise, contrary to the opinion of Dalton, that the small quantity of azote which is found in the gas of marshes is a constituent part of that gas; for this gas, collected at very different periods, always contained the same proportion of azote.

M. Berthollet, having exposed olefiant gas to the action of heat, did not obtain the results announced by Dalton. Far from finding only two compounds between hydrogen and carbon, he found, on the contrary, that they are capable of uniting in indefinite proportions, which vary according to the degree of heat which they have experienced.

M. Berthollet likewise exposed oxycarbureted hydrogen gas to the action of heat, and obtained results analogous to those just mentioned. It deposited carbon, and its specific gravity diminished. Carbonic oxide gas was exposed in a red-hot tube to the action of hydrogen, without undergoing decomposition. This is inconsistent with the opinion of Dalton, who considers oxycarbureted hydrogen as a mixture of carbonic oxide gas and carbureted hydrogen gas; for in order to explain this experiment by the hypothesis of Dalton, we must ascribe all the changes which heat produces upon oxycarbureted gas to the carbureted hydrogen which it contains, which is very difficult, M. Berthollet having proved, by a direct experiment, that hydrogen has no action on carbon.

M. Thenard has made very singular experiments on ammoniacal gas, nearly inexplicable in the present state of chemistry. If we expose this gas in a state of purity to heat in a close porcelain tube, very very little of it undergoes decomposition; but the decomposition goes on very rapidly if we put into the tube iron, copper, silver, gold, or platinum. These metals undergo a change in their physical qualities, but neither increase nor diminish in weight, neither take from nor give out to the gas any thing ponderable. Iron possesses this property in the highest degree. All the other metals (except the five above-mentioned) are destitute of the property altogether. The gas decomposed by this singular method consists of three measures of hydrogen to one of azote. Sulphur and charcoal likewise decompose ammonia; but form with its elements new combinations.

A metal cannot be dissolved in an acid without being oxydated. Sometimes it takes the oxygen from the acid itself, sometimes from water. It sometimes happens that a solution saturated with a metal in an acid, when assisted by heat, is capable of dissolving a new portion of the metal. Proust discovered this to be the case with the nitrate of lead. In this case is it the acid or the oxide which furnishes oxygen to this new portion of metal? M. Proust and Dr. Thomson, who repeated his experiments, thought that the oxygen came from the oxide; from which it would result that the whole of the lead thus dissolved would contain a smaller portion of oxygen, or, in other terms, that it would be less oxydized than the oxide which enters into the common nitrate of lead, which is the yellow oxide.

But M. Chevreul, assistant naturalist to the Museum of Natural History, having again examined this question, found that nitrous gas is disengaged when new lead is dissolved in this way, which could not happen unless the nitric acid lost oxygen; from which this chemist concludes that it is the acid which furnishes oxygen to the new portion of lead, and that the solution is changed from the state of a *nitrate* to that of a *nitrite.* A remarkable property, which serves to distinguish the nitrites of lead from the nitrates, is that of forming in the nitrate of copper a precipitate composed of the hydrate of copper and of lead. By these experiments, M. Chevreul restores to the yellow oxide of lead the rank of *protoxide.*

This chemist has been led to examine, in a general manner, the salts which lead forms with nitric acid. He has shown that there are two nitrates and two nitrites; one of which in each class contains twice as much oxygen as the other. He suspects that there exists a third species of nitrite containing four times less oxide than the first.

Porous bodies absorb gases in different proportions, and charcoal is one of those that absorb the most. The accurate knowledge of the limits of this absorption being important in chemical operations, M. de Saussure has lately examined it with much care and success. All charcoals have not that property in the same degree, and all gases are not absorbed in the same proportion. The same charcoal will absorb 90 times its bulk of ammoniacal gas, and scarcely 1·75 of hydrogen gas.

M. Thenard has repeated these experiments with some variations, and has obtained nearly the same results. He has thrown the whole into the form of a table. He has observed, as Saussure and Count Rumford had done in other experiments, that oxygen gas is changed into into carbonic acid gas, though the temperature be not high. Nitrous gas is partly decomposed, and carbonic acid and azotic gas disengaged. But sulphureted hydrogen is the gas the absorption of which presents the most remarkable phenomena. It is destroyed in a short time, water and sulphur deposited, and so much heat evolved that the temperature of the charcoal is greatly elevated.

M. Lampadius, a German chemist and philosopher, while distilling iron pyrites with charcoal, had obtained a substance, liquid and volatile, the composition of which was doubtful. Lampadius himself, and the late M. Amedée Berthollet, considered it as a compound of sulphur and hydrogen; M. M. Clement and Desormes, as a compound of sulphur and charcoal. M. Clusel, operator of chemistry in the Polytechnic School, wishing to ascertain the nature of this substance, attempted to decompose it by making it pass over plates of copper in hot tubes; but this method not having entirely succeeded, he endeavored to analyse it by means of the Voltaic battery, and after many attempts, delicate and numerous precautions, and a skilful use of different chemical bodies, he conceives that he has determined its composition as follows:

| | |
|---|---|
| Sulphur . . . . . . . . | 59 |
| Charcoal . . . . . . . | 29 |
| Hydrogen . . . . . . . | 6 |
| Azote . . . . . . . . | 7 |
| | 100 |

But he found in his products more sulphur and charcoal than he had employed in his experiment.

M. Thenard resumed the first method of Cluzel, which, being much less complicated, promised more decisive results. By making the liquid of Lampadius pass more slowly over copper in hot tubes, he completely decomposed it into 85 or 86 sulphur, and 14 or 15 charcoal, without either azote or hydrogen.

It will be seen in our preceding reports that M. Delaroche was employed in ascertaining, by new experiments, the phenomena which animals present when exposed to a high temperature. He ascertained that the cutaneous and pulmonary evaporation was one of the causes which prevented animals from assuming completely the temperature of the surrounding medium; but that they did not preserve their own temperature unaltered, as had been said, but became hotter by degress. But it was observed that if the temperature of animals increased as that of the surrounding medium, they ought to reach a still higher temperature, because to that of the medium they ought to join that which is produced by respiration.

M. Delaroche, therefore, wished to determine the difference which the result of respiration, or, in other terms, the absorption of oxygen, would undergo in an air more or less heated, and he found it so small that it is difficult to draw any conclusion. It is in the proportion of five to six. M. Delaroche conceived that there might be no connection between the frequency of respiration and the chemical phenomena of that process; for in a hot air the number of respirations was greatly increased. An interesting remark is, that cold-blooded animals animals show a much greater difference than others, and that heat sensibly increases the activity of their respiration—a fact which may assist us to explain several phenomena of their economy.

The calculi which occasionally form in the gall bladder, and which have hitherto resisted all the skill of the physician, are usually composed of the substance called *adipocire* by chemists, because its characters resemble both those of tallow and of wax. But it appears that they are likewise subject to vary in their nature; for M. Orsila, a doctor of medicine, has analysed some quite different, which contained no adipocire, but were composed of yellow matter, green resin, and a small quantity of picromel.

M. Vauquelin, continuing his researches on vegetable principles, has subjected the *daphne alpina* to numerous experiments. This shrub is known by the excessive acridity of its bark, which is employed in medicine as a rubefacient, and the extract of which mixed with fatty matter forms a pomatum, which in many cases is substituted for that of cantharides. By digesting this bark in alcohol and water he discovered in it two new principles of a very remarkable nature.

The first, which Vauquelin calls the *acrid principle*, is of an oily and resinous nature. Not becoming volatile but at a heat superior to that of boiling alcohol, it does not rise with that liquid, but may be distilled over with water.

The second principle, named *bitter principle*, is soluble in boiling water; and on cooling, shoots into white crystals having the form of needles.

The bark of the *daphne* yielded besides, like that of many other plants, a green resin, a yellow colouring matter, a brown substance containing azote since it yielded ammonia, and salts with a base of potash, of iron, and of lime.

M. Vauquelin terminates his memoir with this important observation, that the acrid and caustic vegetable substances are oily or resinous, and contain no acid, in which respect they agree with poisonous plants. Hence he concludes that we ought to suspect those plants as not fit for eating which contain no acid.

Reaumur had announced more than a century ago, that certain fossile teeth acquired a bluish colour, similar to that of the turquoise, when they are cautiously exposed to a graduated heat. M. Sage having observed that prussic acid is obtained by heating a mixture of potash and of the gelatinous substance of the teeth, and that the magnet attracts iron from the powder of calcined teeth, thinks that the blue colour of the western turquoise is due to a real Prussian blue.

*Mineralogy and Geology.*—The fossil spoils of organised bodies still continue to occupy naturalists.

M. Traullé d'Abbeville has presented to the Class the petrified head of a small cetaceous animal, which appears to have belonged to the whale genus, and which was dug out of the harbour at Antwerp. M. le Comte Dejean, Senator, sent a similar head from the same place to the administration of the Museum of Natural History. Many vertebræ of animals of the same class, and numerous shells, have been found in the same place.

M. Traullé likewise presented a portion of the jaw-bone of a rhinoceros, noceros, found on the sand-hills of the valley of the Somme, in the neighbourhood of Abbeville.

M. Daudebart de Ferussac, a young soldier, transported successively by the duties of his function to the most opposite parts of Europe, took advantage of his leisure moments to examine the fossils; and as he has paid particular attention to land and fresh water shells, he attached himself from choice to that sort of soil discovered in the neighbourhood of Paris by M. M. Brogniart and Cuvier, which containing only fresh water shells, appeared to these naturalists not to owe its origin to the sea, as is the case with most other secondary formations.

M. de Ferussac has observed similar beds containing the same shells, and composed of the same substances, in the south of France, in several provinces of Spain, in Germany, and as far as the bottom of Silesia; so that there can hardly be a doubt that these formations are general.

M. de Ferussac, to give more precision to his observations, has examined the shells themselves, has determined their species with great accuracy, and has given good observations on the variations which they may experience, and several happy ideas respecting the character which may serve to distinguish the genera.

M. Cuvier has just published, in four volumes in 4to. with numerous plates, a collection of all his memoirs on the fossil bones of quadrupeds. He has described 78 species, 49 of which were certainly unknown to naturalists, and 16 or 18 are still doubtful. The other bones found in these recent beds appear to belong to animals known. In a preliminary dissertation, the author explains the method which he followed, and the results which he obtained. It appears to him, from facts which he has established, that the earth has undergone several great and sudden revolutions, the last of which, not more remote than 5 or 6,000 years, destroyed the country at that time inhabited by the species of animals existing, and offered for a habitation to the feeble remains of these species, continents which had been already inhabited by other beings, which a preceding revolution had buried, and which appeared in their actual state at the time of this last revolution.

---

*Fungin.*—This name has been recently given by Braconnet to the fleshy part of mushrooms, which he conceives to be a peculiar vegetable principle; and which, according to him, possesses the following properties:—

It may be obtained pure by boiling it in a weak alkaline solution. In that state it is whitish, soft, insipid, possesses little elasticity, and readily yields to the teeth. It would appear that fungin thus purified may be used as an article of food, from what mushroom soever it has been obtained. The poisonous qualities of mushrooms, it would seem, reside in the juices, not in the fungin. This substance, when dried, burns with considerable splendour, emitting an odour similar to that of burning bread, and leaving behind it a white ash.

Dried fungin, when distilled in a retort, yields about half its weight of of a liquid product, consisting partly of a brown oil, and partly of water, holding a good deal of ammonia in solution. It yields no acid, which distinguishes it very much from wood. The charcoal remaining in the retort amounts to rather more than one-fourth of the dry fungin subjected to distillation. This charcoal exhibited traces of sulphureted hydrogen, and contained sand, phosphate of lime, and traces of carbonate of lime, phosphate of lime, and of alumina.

Fungin does not dissolve in alkaline solutions, in which respect it differs essentially from lignin, which is readily dissolved by a weak alkali; but if fungin be boiled in a very strong alkaline lye it is partly dissolved, and a saponaceous product obtained. Ammonia dissolves a small portion of fungin, and lets it fall again in white flocks when exposed to the air.

Weak sulphuric acid has no action on fungin; but when concentrated, this acid chars it, and at the same time sulphureous acid and vinegar are formed.

Muriatic acid dissolves it very slowly, and converts it into a gelatinous matter. It is thrown down in flocks by the addition of potash to the acid. Chlorine passed over dry fungin suspended in water converts it into a yellow matter, having at first an acrid taste, which it gradually loses by exposure to the air.

When digested in diluted nitric acid, azotic gas is disengaged. Heated with concentrated nitric acid, it swells, and effervesces, at first violently, but the action soon subsides. When the acid is driven off there remains a liquor, containing oxalate of lime, some prussic acid, and two fatty matters, the most abundant similar to tallow, the other to wax. By evaporating the liquid a considerable quantity of oxalic acid in crystals was obtained. The mother water still contained oxalic acid, and a portion of the bitter principle from indigo.

When fungin is steeped in an infusion of nutgalls, it imbibes a considerable portion of the tannin, and acquires a fawn color.

When left to putrefy spontaneously in water, it emitted first the odor of putrefying gluten, then that of putrid meat. Neither acid nor ammonia was found in the water; but it contained a portion of mucilage, which gave it viscosity, and the property of precipitating with acetate of lead. The fungin itself assumed the aspect of gluten, without however possessing its properties.

The individual fungi which have been analyzed, are the following:

1. *Agaricus Volvaceus.*—This fungus was rubbed with water, and the liquor filtered. Distilled vinegar separated some albumen, which principle also coagulated in heating. Litmus was reddened by the liquor. Lime water and muriat of lime precipitated white flocculi: nitrat of lead also produced a phosphate of this metal. On evaporating the juice of this fungus to dryness, and treating the residue with alcohol, this dissolved a substance much resembling sugar, but more easily crystallizable. The prevailing acids of this fungus were the phosphoric and acetic. It also was found to contain fungine albumen, gelatine, and adipocire, besides some other saline matters.

2. *Agaricus Acris.*—This champignon is common in woods, and is white, with a short and thick stalk, and a broad hat-shaped summit; though reckoned poisonous, it is often eaten with impunity; and the author author found that the acrid taste totally disappeared by slight drying. The composition of this fungus much resembles the last mentioned. The *marc* (or cake remaining after all the juice has been separated by water and pressure,) contains a good deal of adipocire or spermaceti-like matter, which may be extracted by boiling alcohol.

3. *Hydnum repandum, Linn.*—This is an acrid fungus, common in woods, which, however, is eaten by the country people in France, boiled with fresh water, salt and pepper. Its chemical analysis shows most of the ingredients of the first-mentioned fungus, but it contains a remarkable quantity of sugar.

4. *Hydnum hybridum* resembles the other species.

5. *Agaricus cantharellus, Linn.* resembles the other species.

6. *Boletus Viscidus.*—This fungus tastes sour; when rubbed with warm water, it dissolves almost entirely into a thick ropy liquid, which retains its clamminess when largely diluted with water. Neither tannin, nor corrosive sublimate, nor lime-water, nor alkalies, produce any obvious change. The ropy liquid just saturated with alkali and evaporated, was found to be (with the exception of acetate of potash) almost identical with animal mucus.

7. *Boletus pseudo-igniarius.*—The expressed juice of this boletus has a yellow color and an agreeable mushroom flavor mixed with a slight acidity. Some albumen was separated by heat, and the liquor evaporated nearly to dryness, and treated with alcohol partly dissolved on it, giving not a concrete sugar, but an uncrystallizable saccharine mucus. On the other hand, the portion of the extract of this boletus, which was insoluble in alcohol, was re-dissolved in water; nitrat of lead was added, which gave an abundant precipitate, from which the lead was separated by sulphureted hydrogen, leaving in the liquor the acid contents of the fungus. Part of this liquor formed a crystallized acid or evaporation, and the residue was an acid deliquescent mass, which was phosphoric acid.

The crystallized acid was examined with care, being different from any one hitherto known. To purify it, it was dissolved in alcohol, and by evaporating the spirit the pure acid was left in the form of white permanent prismatic grains, feeling hard in the mouth and giving a taste like cream of tartar. This acid forms a salt with lead, which is somewhat soluble; it precipitates iron from its solutions when fully oxydated, but not in an inferior state of oxydation. Lime-water and barytes produce but little change. This acid, when heated by itself in a retort, sublimes in crystalline needles, which are the acid unaltered, except being rendered purer, by parting with a portion of acrid oil in the process. This acid forms with ammonia, a salt of sparing solubility, which readily and totally decomposes the solutions of highly oxydized iron, but does not alter the sulphats of lime, alumine, and manganese; and hence it may be employed like the succinic or benzoic acids, in the hitherto difficult process of separating iron alone from various compound solutions. On the whole, the concrete acid of this boletus, has the nearest resemblance to the benzoic in its properties.

8. *Mucor Septicus.*—This fungus generally appears on the bark of hot-beds, in the form of a yellowish cake, in three distinct laminæ,

 the the upper one of which is usually covered with an effervescence of carbonat of lime; and this earth is obtained abundantly from the whole fungus, so that it effervesces violently in acids. The author obtained from it also the substance he has described under the term fungine and albumen. Another singular product of this fungus, is a yellow powder, which abounds in the fungus when gathered and slowly dried. This powder is not readily soluble in water alone, or in alcohol; but a weak alkaline lye extracts it in abundance, and acids separate it of a blood red color. This matter seems to have a strong affinity for wool and silk, and gives a good dye.

9. *Phallus Impudicus.*—This very fetid and short-lived fungus, gives most of the usual constituents common to all the other fungi.—*Annales de Chimie.*

---

*New Salts of Lead.*—Chevreul has lately examined some salts of lead which had been originally discovered by M. Proust, and in which he had conceived that there existed an oxide containing less oxygen than the yellow oxide. I afterwards examined the same salts, and found that they contained the common yellow oxide. Chevreul has found that the acid combined with the oxide in these salts is not, as we had supposed, the *nitrate*, but the *nitrous* acid. This is what distinguishes them from the nitrates of lead formerly known. I shall here give a sketch of the results obtained by Chevreul.

1. There are two nitrates of lead. 1. The common octahedral nitrate, which is a supernitrate, containing an excess of acid. 2. The neutral nitrate, composed of scales. It may be formed by boiling together a mixture of yellow oxide of lead and octahedral nitrate.

The supernitrate of lead is composed of

| | | |
|---|---|---|
| Acid . . . . . | 33 . . . . . | 100 |
| Yellow oxide . | 67 . . . . . | 203 |

The scaly or neutral nitrate is composed of

| | | |
|---|---|---|
| Acid . . . . | 19·86 . . . . . | 100 |
| Yellow oxide | 80·14 . . . . . | 403 |

So that the quantity of oxide in the neutral salt is twice as great as in the supersalt.

2. Three hundred and fifty parts of water, 4 parts of supernitrate of lead, and 6 parts of lead, were boiled in a flask for 14 hours: 5·4 parts of the lead were dissolved. The liquid became at first yellow, but the yellow colour gradually disappeared, and a greyish white matter precipitated. The glass was attacked. The grey matter consisted of a mixture of silica and hydrate of lead. The lead amounted to 0·47 of a part of yellow oxide.

3. The liquid deposited needle-form crystals, which weighed 5·95. The mother water contained nitrate of potash.

4. By this process the nitric acid is decomposed, and converted into nitrous acid and nitrous gas. Two salts are formed, one in plates (a nitrite), and one in needles (a subnitrite).

 5. The

5. The nitrite is best prepared by causing a current of carbonic acid gas to pass through the solution of the subnitrite.

6. The nitrite possesses the following properties: It is little soluble in cold water; but boiling water dissolves nearly one-tenth of its weight of it. It was decomposed by all the acids tried. Even carbonic acid partially decomposes it, converting it into subnitrite. Its constituents are,

| | | |
|---|---|---|
| Acid . . . | 18·15 . . . . . | 100 |
| Yellow oxide | 81·85 . . . . . | 450 |

7. The subnitrite possesess the following properties. The colour is reddish yellow. It crystallizes in needles: 100 parts of boiling water dissolve about three parts of it, and retain about one part when cooled down to 73°. It is composed of

| | | |
|---|---|---|
| Acid . . . | 9·9 . . . . . | 100 |
| Yellow oxide | 90·1 . . . . . | 910 |

So that it contains double the quantity of oxide that exists in the nitrite. See Ann. du Museum d'Histoire Naturelle, N° III. p. 1888.

There is one circumstance in the above statement obviously inaccurate. Chevreul says, that by the action of the lead the nitric acid was resolved into nitrous acid and nitrous gas. Such a resolution is impossible; because nitric acid contains more oxygen than exists in nitrous acid and nitrous gas. The meaning, however, may be made out, though it is obscurely expressed. What Chevreul must mean is obviously this: the nitric acid gave out a portion of its oxygen to the lead, and the remainder was resolved into nitrous acid and nitrous gas—an effect which might very well happen.

---

*Meteorological Observations in April, made at Tottenham by* Mr. Howard.

1. Stormy, with rain.—2. Hoar frost: a sprinkling of opake hail about sun-rise; several showers of this, and some rain, during the day.—3. Hoar frost: *Cumulus* a. m. showers of snow and of opake hail p. m.—5. a. m. *Cirrostratus:* wet and windy.—10. *Cirrus* and *Cumulus* clouds: the winds increase in strength from the N.E. The mornings have been misty of late, and there have been plentiful dews, in consequence of the great difference between the temperature of day and night.—15. Wind boisterous in the evening.—16. Cloudy a.m.—17. Slight showers.—20. From the 7th of this month we have had summer-like days and cold nights; the roads have become very dusty, and the earth considerably dry.—21. Some clouds of a threatening appearance from the N.E. in the evening, attended with depression of temperature.—22. p. m. Hasty showers, mixed with hail; after which, steady small rain till evening.—23. Cloudy: several scanty hail showers, from large *Nimbus* clouds passing over. During the approach of one of these, a slender, tapering, and somewhat twisted column appeared in front, detached from the main body, and reaching down to the earth, in the manner of of a water-spout. In a few minutes, by spreading on all sides, it became incorporated with the rest of the shower. This is not a very uncommon appearance; but I have seldom seen it so perfectly exhibited.

*New Patents.*

William Mitchell, surgeon, late in Ayr, now in Edinburgh; for an important discovery in the manufacture of soap. Dated March 3, 1813.

Richard Edwards, of the parish of Budock, in the county of Cornwall, Doctor of Physic, and William Williams, of the borough of Penryn, in the same county, surgeon; for a process for extracting arsenic from any of the ores, or other substances in which it is contained, in a purer state than it is at present procured in this kingdom. Dated March 15, 1813.

William Robert Wale King, of Union-court, Holborn-hill, in the city of London, tin-plate worker; for certain improvements in the application of heat to the purposes of boiling water and other fluids, and to other useful purposes, and of the apparatus for performing the same. Dated March 22, 1813.

*Deaths by Small-pox, from the Weekly Bills.*

Quarterly amount from December 22d to March 9th, 319.

| | | |
|---|---|---|
| March | 16, . . . . . . . . | 16 |
| | 23, . . . . . . . . | 11 |
| | 30, . . . . . . . . | 14 |
| April | 6, . . . . . . . . | 6 |
| | 13, . . . . . . . . | 8 |
| | 20, . . . . . . . . | 9 |
| | 27, . . . . . . . . | 15 |
| May | 4, . . . . . . . . | 8 |

On Sunday, the 4th of April, died, at his house in Bartlett's-buildings, Holborn, at the age of 71, Dr. Andrew Marshal, a Member of the Royal College of Physicians, and for many years a distinguished teacher of anatomy in London. Besides being a most minute anatomist, he was a profound scholar, and an able and successful practitioner. We understand that he has left behind him a large number of valuable manuscripts, on various interesting subjects of Pathology, the fruits of his own experience and observation, and which he has bequeathed to the care of his intimate friend Mr. Sawrey, of Chancery-lane, by whom it is expected they will in due time be communicated to the public.

Mr. Richard Williams, of Aberystwith, one of the dressers to Mr. Cline at St. Thomas's Hospital, has been awarded the first prize given by the Professors of Anatomy at that Theatre, for the best Anatomical tomical Preparation made there during the last winter. The same young gentleman is engaged on a small work on Scrofula.

---

In the course of the present month will be published, Practical Remarks on Diseases resembling Syphilis, with Cases; by John Whitsed, surgeon, Peterborough, Member of the Royal College of Surgeons, London.

---

*Medical and Chemical Lectures and Examinations.*—Dr. Hooper and Dr. Ager will commence their next Course on the Theory and Practice of Physic, Chemistry, and the Materia Medica, at the Theatre, 26, Cork-street, Burlington Gardens, on Monday, June 7th, 1813, at eight o'clock in the morning.

Three Courses are given yearly, beginning in February, June, and October, each on the first Monday of the month.

---

Mr. Thomson will commence his usual Course of Lectures on the Science of Botany on Saturday the 12th of June next, at two o'clock in the afternoon, at which hour a Lecture will be delivered every day except Sunday until the Course be finished. For the convenience of those gentlemen who attend the medical classes, the Lectures will be delivered in Mr. Brooks's Anatomical Theatre, Blenheim-street.

This course embraces both the Principles of *Phytology*, or the Physiology of Vegetables, and *Systematic Botany;* and particular care is taken to select as specimens and illustrations, those plants, chiefly, which are used in medicine and the arts.

A syllabus of the lectures may be had of Mr. Callow, Medical Bookseller, Crown-court, Princes'-street; or of Mr. Thomson, at his house, No. 92, Sloane-street, any morning before ten o'clock, or evening after six o'clock.

---

Dr. Squire will, on Tuesday the 8th of June, begin a Course of Lectures on the Theory and Practice of Midwifery, and the Diseases of Women and Children.—Particulars may be known at Dr. Squire's house, Ely-place, Holborn.

---

Dr. Ramsbotham will read a Course of Lectures on the Science and Practice of Midwifery, during the summer season, at his house, No. 9, Old Jewry. The course will be commenced on Monday, June 7th, at half after seven in the morning.

---

*Theatre of Anatomy, Dean-street, Soho.*—Mr. Carpue will recommence his Lectures on the 10th of June.

METEO-

